# Aggressive Angiomyxoma of the Vulva: A Précis for Primary Care Providers

**DOI:** 10.1155/2013/183725

**Published:** 2013-08-13

**Authors:** R. Elkattah, O. Sarkodie, H. Otteno, A. Fletcher

**Affiliations:** ^1^Department of Obstetrics and Gynecology, Quillen College of Medicine, East Tennessee State University, 325 N State of Franklin Road, Johnson City, TN 37604, USA; ^2^Department of Pathology, Quillen College of Medicine, Box 70568, East Tennessee State University, Johnson City, TN 37614, USA

## Abstract

Vulvar aggressive angiomyxoma (AA) is a rare mesenchymal tumor of the vulva. Due to its slow-growing nature, it is often overlooked and misdiagnosed by primary care providers (PCPs). We describe a case report of vulvar AA in a 38-year-old woman who underwent complete surgical excision of the neoplasm with no evidence of recurrence on a 5-year followup. A literature review follows to provide PCPs with the clinical, radiologic, and pathologic features that this tumor displays.

## 1. Case Illustration

**Figure 1:**
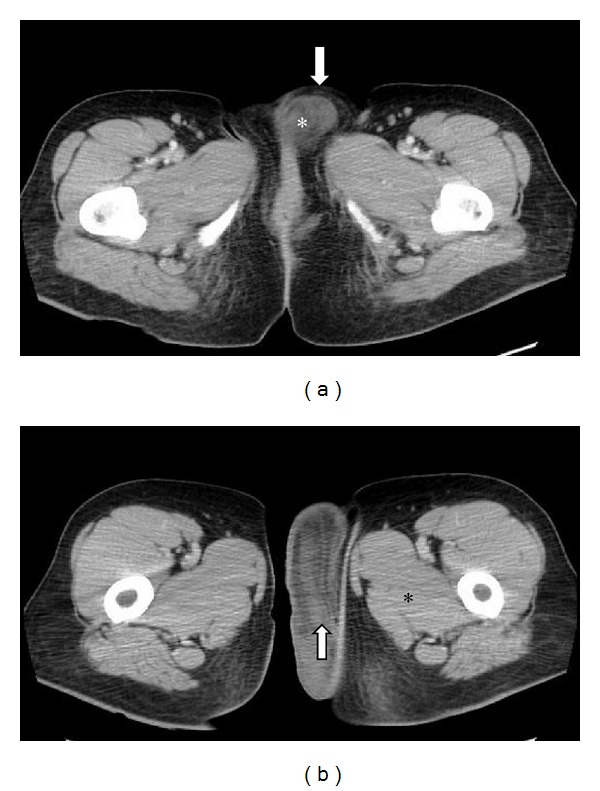
(a) Axial noncontrast CT image showing vulvar skin edema (arrow) and the suspected aggressive angiomyxoma at its anterior origin. (b) Axial CT image with contrast showing the posterior extent of the aggressive angiomyxoma with the swirled appearance (arrow) and the attenuation difference between it and surrounding skeletal muscles; less attenuation compared to skeletal muscle (asterisk).

**Figure 2:**
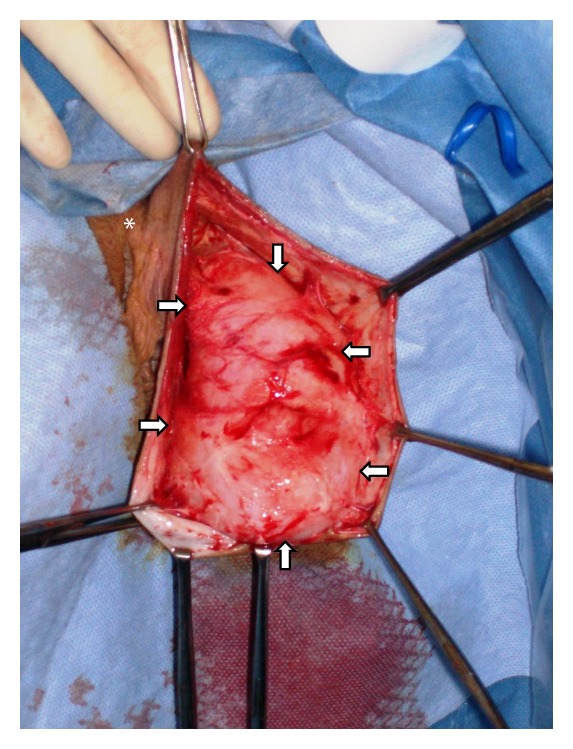
Intraoperative image delineating the borders of the vulvar AA (arrows) and the clitoral hood (asterisk).

**Figure 3:**
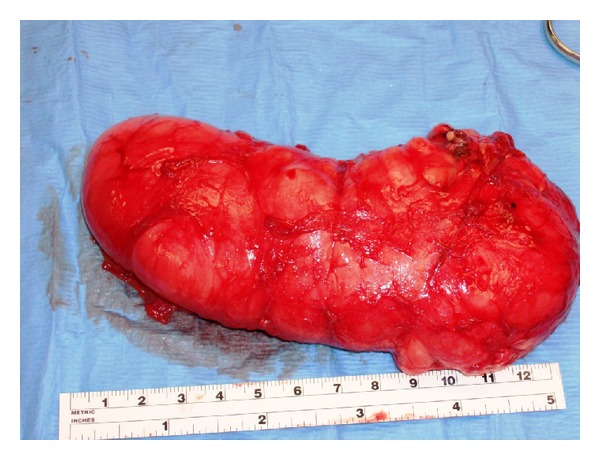
Gross vulvar AA: well-circumscribed and well-encapsulated fleshy tan gelatinous mass.

**Figure 4:**
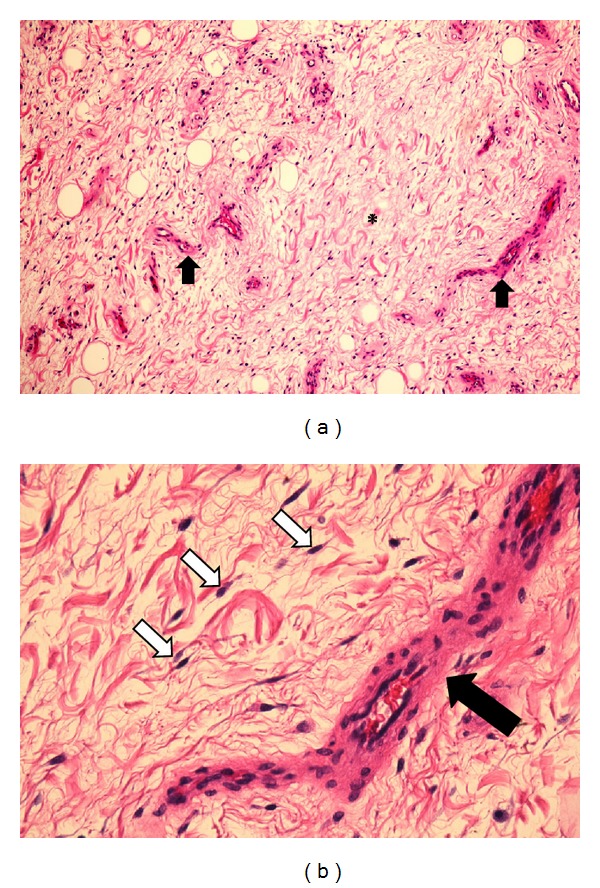
(a) Low-power (10x) H&E slide showing widely scattered bland spindle cells within a myxoid background (asterisk) with accompanying vascular structures (arrows). (b) High-power (40x) H&E slide showing bland spindle cells (white arrows) scattered within a myxoid background and nearby vascular structures (black arrow).

Our patient is a previously healthy 38-year-old, G2P2, Caucasian female. Her past medical history includes two cesarean deliveries, a tubal ligation, appendectomy, and excision of a benign breast tumor. She denied any family history of gynecologic cancers and any personal history of venereal disease. Her gynecologic history is otherwise normal. She had presented for her annual checkup with complaints of painless swelling of the left labia majora that had been progressively worsening over the past 3 years ever since her last cesarean delivery. She had raised this concern to several primary care providers (PCPs) and with previous physical examinations, she was reassured that it was likely postoperative labial swelling that followed the cesarean delivery and would likely resolve with time. Upon interviewing her, she reported numbness of the skin overlying the swollen area and denied any discharge, pruritis, pain to palpation, or any urinary symptoms. She did however complain of progressively worsening dyspareunia and consequently affected her quality of life. Gynecologic examination showed an enlarged left labia majora. It contained an elongated, soft, nonmobile, nontender, sausage-like mass measuring approximately 12 × 6 × 4 centimeters extending longitudinally between the mons pubis and the anal verge along the left labial fold. There was no evidence of any overlying skin infection, erythema, or drainage. Her physical exam was otherwise within normal limits. Abdominal and pelvic computed tomography (CT) with oral and intravenous contrast showed left labial edema, no ascites, and no inguinal or pelvic lymphadenopathy. This lesion was localized within the vulva and did not extend beyond the pelvic diaphragm (Figure 1). The CT scan could not exclude a vulvar mass. The differential diagnosis at this juncture included a labial neoplasm, cyst of the canal of Nuck (abnormal outpouching of the peritoneum extending into the labia majora), and a hernia. The decision to surgically explore this suspected lesion was taken. Once the skin was dissected away, it was evident that this lesion was a mass. The patient had an uneventful intraoperative course with complete excision of this mass (Figure 2) and an estimated blood loss of 150cc. On gross examination, the mass comprised of soft tissue measuring up to 16 cm in diameter and was well circumscribed (Figure 3). The cut surface showed a well-encapsulated fleshy tan gelatinous mass. Intraoperative frozen section of the specimen showed a spindle cell soft tissue tumor. The final pathology using H&E staining showed widely scattered bland spindle cells with ill-defined cytoplasm within a myxoid background with accompanying thin and thick-walled vascular structures all of which are characteristic of an aggressive angiomyxoma (Figure 4). A second opinion at Mayo Clinic confirmed the finding. The postoperative course was uneventful. The patient had complete resolution of the swelling and remains asymptomatic 5 years later.

## 2. Background

First described in 1983 by Steeper and Rosai [1], perineal aggressive angiomyxomas (AA) are rare mesenchymal tumors. There are at least 150 reported cases in the medical literature [2] the vast majority of which afflict premenopausal females in their 3rd and 4th decades of life, with a female-to-male ratio of 6.6 : 1 [2, 3]. The term *“aggressive” *refers to the locally infiltrative nature of this lesion and its tendency to recur [4]. Although slow growing, they may attain large sizes (up to 60 cm) but are usually 10–20 cm in their greatest diameter [2] and have a marked tendency to recur locally (up to 72% [3]), even decades after resection [4]. With an overall good prognosis, they have a low metastatic capacity [3, 4]; with only two cases of pulmonary metastasis reported following recurrent AA, one of which was fatal [2, 5]. AA have been reported to invade pelvic organs and bone; however they typically exert a mass effect and displace normal tissues [3].

The pathogenesis of vulvar AA is poorly understood; however genetic alterations along the 12q chromosome region have been implicated [6]. Macroscopically, AA are bulky, soft, homogenous tissue masses which are easily compressible and fleshy in consistency [3, 7] and exhibit a gelatinous appearance on cut section [2]. They are usually not encapsulated and typically have finger-like projections that extend into neighboring tissues [2, 3]. Microscopically, the tumor is rather hypocellular and made of a mixture of spindle cells in a loose myxoid stroma composed of collagen fibrils and infiltrated by numerous blood vessels of varying caliber [2, 3, 8]. Mitotic figures and nuclear atypia are almost always absent [8]. On an ultrastructural level, AA arise from neoplastic fibroblastic cells [9]. Immunohistochemically, most AA express different combinations of estrogen and progesterone receptors, vimentin, desmin, smooth-muscle actin, muscle-specific action, CD34, and CD44, but all are invariably negative for S-100, CEA, and keratin [2, 3, 8, 9].

Several imaging modalities have been used in identifying and describing AA. Sonography usually reveals a hypoechoic or cystic mass [10]. CT scan typically reveals a tumor with well-defined margins with attenuation less than that of muscle, likely related to the loose myxoid stroma and high water content of this tumor [10]. Relative to muscle signals on magnetic resonance (MR) imaging, AA shows an isointense signal on T1-weighted images and a hyperintense one on T2-weighted images [10]. A swirled appearance on CT and MR imaging is often present in 83% of cases as well [10]. Angiography typically shows a hypervascular mass [2, 3, 10]. It is agreed that CT and MR imaging modalities are superior in determining the pelvic transdiaphragmatic extent of AA lesions and are important imaging modalities for surgical decision making [3, 10].

Multiple treatment modalities have been described; however complete surgical excision—when possible—should be sought. Partial excision may be warranted when high operative morbidity is anticipated [3]. Unfortunately, recurrence may occur even with negative surgical margins [11]. This may necessitate multimodal therapies using surgical and medical means to treat recurrent AA [2]. Adjunct hormonal treatment with tamoxifen, raloxifene, and gonadotropin-releasing hormone analogs (GnRHa) has been described with varying degrees of success ranging from noresponse to complete remission of primary and recurrent AA [2, 3, 12]. This has been attributed to the fact that many of these tumors express estrogen and progesterone receptors and are sensitive to hormonal therapy. Furthermore, preoperative shrinking of tumors using GnRHa might increase chances for complete excision and minimize the radicality of surgery [8]. Radiotherapy and chemotherapy have been used as adjunctive therapies but are unlikely to be useful as AA has low mitotic activity [2, 3, 8]. Arterial embolization has been reported but is usually not performed as these tumors are supplied by several feeding vessels [2, 3, 11]. Due to its rarity, the role of sentinel lymph node biopsy and lymphadenectomy in AA is yet to be determined. Despite this wide array of treatment options, recurrence of AA is reported to be as high as 72% [3].

## 3. Discussion

Vulvar AA is a rare and intriguing finding when encountered in a primary-care setting. Misdiagnosis occurs in more than 80% of cases [3, 13], primarily due to the rarity of this entity, and its vast differential diagnosis. Clinically, the differential diagnosis of a perineal mass in females includes a vulvar abscess/neoplasm/cyst, Bartholin abscess, Gartner's duct cyst, vaginal cyst/polyp, vulvar edema, lipoma, canal of Nuck hernia, pelvic floor hernia, and vaginal prolapse [3, 8, 12, 14]. Histopathologic mimics of AA include angiomyofibroblastoma, fibroma, myxofibrosarcoma, myxoid leiomyoma, lymphangioma, neurofibroma, malignant mesenchymoma, malignant fibrous histiocytoma, myxolipoma, sclerosing hemangioma, botryoid pseudosarcoma, myxoid smooth muscle and nerve sheath tumors, mixed mesodermal tumor, leiomyosarcoma, and embryonal rhabdomyosarcomas [2, 3]. History taking and physical exam further guide the clinical workup. Imaging is ultimately recommended, particularly CT and MR imaging, to identify the extent of pelvic involvement of perineal masses as their sizes are often underestimated by clinical examination [2, 3, 10]. When a vulvar lesion does not traverse the pelvic floor, a perineal approach in surgery is attempted. On the other hand, when the pelvis and pelvic floor are involved, a concomitant abdominoperineal approach would be indicated [3]. In our patient, physical exam revealed a well-circumscribed fleshy mass in the left labia majora, and CT scan confirmed the superficial nature of this lesion with respect to the pelvic diaphragm thus limiting our differential diagnosis to an unspecified labial neoplasm, cyst of the canal of Nuck, and a hernia. This provided crucial guidance in selecting a proper surgical route and ultimately in optimizing the exploration and treatment of this particular finding.

Unlike most AA, our case was encapsulated by a fibrofatty layer, and there were no evident finger-like projections into neighboring tissues. Furthermore, the excised lesion had negative margins on pathologic evaluation. This may explain why there has been no recurrence in the last 5 years. On H&E staining, the tissue resembled a typical aggressive angiomyxoma, obviating the need for any further immunohistochemical analysis.

Our patient required no additional treatment or any imaging postoperatively and remained asymptomatic throughout the subsequent 5 years after her surgery. Despite these facts, AA is notorious for local recurrence in approximately 70% of the cases after a period of 2 years postoperatively [7] and has been reported 20 years postoperatively as well [4]. Han-Guerts et al. [15] propose the following guideline in treating AA: (1) complete excision of the lesion when possible and avoiding mutilating surgery, (2) adjunct therapy when partial resection is performed is acceptable using arterial embolization and/or hormonal treatment, and (3) radiotherapy is reserved to cases that are resistant to embolization and/or hormonal therapy and still symptomatic. Multimodal therapy is advised when complete resection of the AA is not possible.

There are no guidelines in the postoperative management of vulvar AA; however due to the high recurrence rate and potential morbidity associated with undiagnosed recurrences, several authors have recommended periodic evaluations with physical examination and MR imaging up to 15 years after treatment [2, 12].

## 4. Conclusion

This case report illustrates the challenges that primary care physicians might face with vulvar masses. AA is a rare entity but should always be considered especially when it presents in an insidious painless fashion, particularly in premenopausal females in their 3rd-4th decades of life. PCPs should maintain a high index of suspicion and should perform a systematic clinical examination of the perineal mass. If a perineal mass is suspected, pathologic evaluation and radiographic studies using MR imaging or CT scan should help in reducing the number of misdiagnosed cases of AA preoperatively. Once its anatomical location is defined, any perineal mass—particularly AA—can be optimally treated by surgical excision while avoiding mutilating surgery. When complete resection is possible, it should be sought as it offers the lowest recurrence rate. AA is rarely life threatening and therefore partial resection is acceptable when high operative morbidity is anticipated. Regardless of whether the treatment is surgical, hormonal, or multimodal, it is clear that AA requires close and long-term followup.

## References

[1] Steeper TA, Rosai J (1983). Aggressive angiomyxoma of the female pelvis and perineum. Report of nine cases of a distinctive type of gynecologic soft-tissue neoplasm. *American Journal of Surgical Pathology*.

[2] Dahiya K, Jain S, Duhan N, Nanda S, Kundu P (2011). Aggressive angiomyxoma of vulva and vagina: a series of three cases and review of literature. *Archives of Gynecology and Obstetrics*.

[3] Dierickx I, Deraedt K, Poppe W, Verguts J (2008). Aggressive angiomyxoma of the vulva: a case report and review of literature. *Archives of Gynecology and Obstetrics*.

[4] Kiran G, Yancar S, Sayar H, Kiran H, Coskun A, Arikan DC (2013). Late recurrence of aggressive angiomyxoma of the vulva. *Journal of Lower Genital Tract Disease*.

[5] Blandamura S, Cruz J, Vergara LF, Puerto IM, Ninfo V (2003). Aggressive angiomyxoma: a second case of metastasis with patient’s death. *Human Pathology*.

[6] Nucci MR, Fletcher CDM (2000). Vulvovaginal soft tissue tumours: update and review. *Histopathology*.

[7] Abu JI, Bamford WM, Malin G, Brown L, Davies Q, Ireland D (2005). Aggressive angiomyxoma of the perineum. *International Journal of Gynecological Cancer*.

[8] Sun NX, Li W (2010). Aggressive angiomyxoma of the vulva: case report and literature review. *Journal of International Medical Research*.

[9] Begin LR, Clement PB, Kirk ME (1985). Aggresive angiomyxoma of pelvic soft parts: a clinicopathologic study of nine cases. *Human Pathology*.

[10] Outwater EK, Marchetto BE, Wagner BJ, Siegelman ES (1999). Aggressive angiomyxoma: findings on CT and MR imaging. *American Journal of Roentgenology*.

[11] Chan IM, Hon E, Ngai SW, Ng TY, Wong LC (2000). Aggressive angiomyxoma in females: is radical resection the only option?. *Acta Obstetricia et Gynecologica Scandinavica*.

[12] Fine BA, Munoz AK, Litz CE, Gershenson DM (2001). Primary medical management of recurrent aggressive angiomyxoma of the vulva with a gonadotropin-releasing hormone agonist. *Gynecologic Oncology*.

[13] Güngör T, Zengeroğlu S, Kaleli A, Kuzey GM (2004). Aggressive angiomyxoma of the vulva and vagina—a common problem: misdiagnosis. *European Journal of Obstetrics Gynecology and Reproductive Biology*.

[14] Dragoumis K, Drevelengas A, Chatzigeorgiou K (2005). Aggressive angiomyxoma of the vulva extending into the pelvis: report of two cases. *Journal of Obstetrics and Gynaecology Research*.

[15] Han-Geurts IJM, van Geel AN, van Doorn L, M. den Bakker MDB, Eggermont AMM, Verhoef C (2006). Aggressive angiomyxoma: multimodality treatments can avoid mutilating surgery. *European Journal of Surgical Oncology*.

